# Protective role of testis-specific peroxiredoxin 4 against cellular oxidative stress

**DOI:** 10.3164/jcbn.16-96

**Published:** 2017-05-01

**Authors:** Eisuke Tasaki, Shotaro Matsumoto, Hisashi Tada, Toshihiro Kurahashi, Xuhong Zhang, Junichi Fujii, Toshihiko Utsumi, Yoshihito Iuchi

**Affiliations:** 1 Department of Applied Bioresources Chemistry, The United Graduate School of Agriculture, Tottori University, 4-101 Koyamacho-minami, Tottori 680-8553, Japan; 2 Department of Biological Chemistry, Faculty of Agriculture, Yamaguchi University, 1677-1 Yoshida, Yamaguchi 753-8515, Japan; 3 Department of Pathology and Cell Regulation, Graduate School of Medical Science, Kyoto Prefectural University of Medicine, 465 Kajii-cho, Kawaramachi-Hirokoji, Kamigyo-ku, Kyoto 602-8566, Japan; 4 Department of Biochemistry and Molecular Biology, Graduate School of Medical Science, Yamagata University, 2-2-2 Iidanishi, Yamagata 990-9585, Japan; 5 Graduate School of Sciences and Technology for Innovation, Yamaguchi University, 1677-1 Yoshida, Yamaguchi 753-8515, Japan

**Keywords:** antioxidant activity, fluorescence image, oxidative stress, peroxiredoxin, ROS detection

## Abstract

Peroxiredoxin (PRDX), a newly discovered antioxidant enzyme, has an important role in hydrogen peroxide reduction. Among six PRDX genes (PRDX1–6) in mammals, PRDX4 gene is alternatively spliced to produce the somatic cell form (PRDX4) and the testis specific form (PRDX4t). In our previous study, PRDX4 knockout mice displayed testicular atrophy with an increase in cell death due to oxidative stress. However, the antioxidant function of PRDX4t is unknown. In this study, we demonstrate that PRDX4t plays a protective role against oxidative stress in the mammalian cell line HEK293T. The PRDX4t-EGFP plasmid was transferred into HEK293T cells; protein expression was confirmed in the cytoplasm. To determine the protective role of PRDX4t in cells, we performed image-based analysis of PRDX4t-EGFP expressed cells exposed to UV irradiation and hydrogen peroxide using fluorescent probe CellROX. Our results suggested that PRDX4t-EGFP expressed cells had reduced levels of oxidative stress compared with cells that express only EGFP. This study highlights that PRDX4t plays an important role in cellular antioxidant defense.

## Introduction

Antioxidant enzymes, such as superoxide dismutase (SOD), glutathione peroxidase (GPx), and catalase, play protective roles against oxidative stress caused by elevated reactive oxygen species (ROS), as well as antioxidants.^(1)^ Peroxiredoxins (PRDXs) have attracted attention in recent years as a new family of thiol-specific antioxidant proteins.^(2)^ Six distinct genes comprise the mammalian PRDX family and have been divided into three PRDX subtypes; four typical 2-Cys PRDX, one atypical 2-Cys PRDX, and one 1-Cys PRDX.^(3)^ The major functions of these PRDXs include thioredoxin (Trx)-dependent peroxidase activity,^(2)^ modulation of intracellular signaling through hydrogen peroxide (H_2_O_2_) as a second messenger, and regulation of cell proliferation.^(4–6)^ Although other divergent biological functions have been reported for individual PRDX members, the detailed antioxidant function of PRDX family members remains unknown.

Among mammalian PRDX family members, PRDX4 demonstrates unique properties. Two types of PRDX4 are alternatively transcribed from the single PRDX4 gene, somatic cell type PRDX4 and testis specific PRDX4t. The two forms of PRDX4 differ only in the *N*-terminal sequence derived from exon 1 (exon 1 and exon 1t, respectively) and share the sequences encoded by exons 2–7 (the catalytic center is in exon 3).^(7)^ It has been reported that PRDX4 is primarily located in the endoplasmic reticulum (ER)/ Golgi apparatus, in spite of the presence of a secretory signal sequence encoded by Ex1.^(8)^ PRDX4 plays an important role in regulating disulfide bond formation in proteins and protecting cells from ER stress by metabolizing hydrogen peroxide.^(7,9)^ On the other hand, PRDX4t is entirely restricted to testicular cells, with induction that is sexual maturation-dependent. Surprisingly, PRDX4 gene knockout mice have indicated that expression of PRDX4t decreases compared to wild type mice, and that testicular atrophy and increased cell death is due to oxidative stress.^(10)^ Therefore, we hypothesized that PRDX4t protects cells from ROS-induced damage; however, the cellular antioxidant function is poorly understood.

In this study, we show that PRDX4t contributes to the suppression and scavenging of ROS in mammalian HEK293T cells by using fluorescent microscopy and image-based cytometry. Our findings indicate that PRDX4t actually plays a protective role against oxidative stress in mammalian cells.

## Materials and Methods

### Cell culture and transfection

HEK293T cells were cultured in Dulbecco’s Modified Eagle Medium (DMEM; Thermo Fisher Scientific, Waltham, MA) with 10% fetal bovine serum (v/v; FBS). Cells were incubated at 37°C in a carbon dioxide (CO_2_) gas incubator (Waken B Tech Co., Ltd., Japan) with 5% CO_2_. Mouse PRDX4t cDNA (Ensembl Transcript ID: ENSMUST00000130349.2) was subcloned into the pEGFP-N1 vector (Clontech, Takara Bio Inc., Japan) and was named PRDX4t-EGFP. HEK293T cells were transfected with the PRDX4t-EGFP plasmid or the EGFP (pEGFP-N1) plasmid using the Lipofectamine reagent (Invitrogen, Thermo Fisher Scientific) according to the manufacturer’s manual. Briefly, 1.5 ml tubes containing 100 µl OPTI (Gibco, Thermo Fisher Scientific), 1 µl plus reagent (Invitrogen), and 2 µg PRDX4t-EGFP plasmid or EGFP plasmid, respectively, were prepared and incubated for 5 min at 25°C. After that, 100 µl OPTI and 2.5 µl lipofectamine agent were added to each tube, and tubes were incubated for 30 min at 37°C. After 24 h incubation, HEK293T cells were washed by OPTI-MEM (Gibco, Thermo Fisher Scientific), and incubated with reagent mixed by 800 µl OPTI-MEM added to the cell dishes. After 5 h incubation at 37°C, dishes were treated with 1 ml DMEM containing 20% FBS (final FBS concentration 10%; v/v) and incubated overnight at 37°C.

### Immunoblot analysis

HEK293T cells transfected with the PRDX4t-EGFP or EGFP plasmid were washed three times with PBS and lysed in buffer (20 mM Tris-HCl, 2% protease inhibitor cocktail; v/v), followed by centrifugation at 17,000 *g* for 10 min. Protein concentrations of the supernatant were determined using a BCA protein assay kit (Thermo Fisher Scientific). Cell fractionation was performed using the ProteoExtract subcellular proteome extraction kit (Calbiochem, Merck, Darmstadt, Germany) followed by concentration using a common methanol/chloroform protein precipitation method. SDS-PAGE was perform with 10% polyacrylamide gels (w/v); separated proteins were transferred to polyvinylidene fluoride (PVDF) membranes (AmershamHybond P; GE Healthcare, Little Chalfont, UK), blocked for 2 h in 1% skim milk in TBST (w/v; 0.1% TBS and 0.05% Tween-20), and probed overnight at 4°C with polyclonal anti-rat/anti-mouse PRDX4 antibody.^(10)^ After binding of the appropriate HRP conjugate anti-rabbit IgG antibody (Santa Cruz Biotechnology Inc., Santa Cruz, CA), the ECL plus western blotting detection system (GE Healthcare) was used. Results are shown as one representative experiment.

### PRDX activity assay

Harvested cells were washed twice with PBS and homogenized by sonication in tubes with buffer (20 mM Tris-HCl, 2% protease inhibitor cocktail; v/v), followed by centrifugation at 17,000 *g* for 10 min at 4°C. Supernatants containing proteins were transferred to new tubes and used for experiments as samples. Each sample was measured for protein concentration using the BCA protein assay kit before the extractions. PRDX activity was determined using an indirect assay that links PRDX-mediated oxidation of thioredoxin (Trx) with the recycled reduction of Trx_ox_ (-S-S-) to Trx_red_ (-SH) by TrxR (thioredoxin reductase) using NADPH as the reductant. Quantification of the PRDX activity was assayed by measuring the decomposition of NADPH by monitoring absorbance at 340 nm at 37°C for 10 min. The reaction was started by the addition of the reaction buffer containing 200 µM NADPH, 1.5 µM yTrx, 0.8 µM yTrxR, 50 mM Hepes-NaOH buffer (pH 7.0), and 1 mM EDTA to 100 µg total protein following addition of 100 µM H_2_O_2_. The PRDX activity was defined as the rate of disappearance of NADPH, and we calculated arbitrary units relative to the value from the control.

### Detection of reactive oxygen species (ROS)

ROS were detected using the cell-permeable, peroxide-sensitive probes, CellROX Orange Reagent and CellROX Deep Red Reagent (Invitrogen) according to the manufacturer’s instructions. The dye exhibits bright orange fluorescence upon oxidation by ROS. We prepared HEK293T cells transduced with blank, PRDX4t-EGFP plasmid, or EGFP plasmid for 24 h. For H_2_O_2_ stress assays, cells were incubated with 5 µM CellROX Orange reagent in PBS for 30 min; 250 µM H_2_O_2_ was added after 15 min of treatment. For UV irradiation stress assays, cells were incubated with 5 µM CellROX Orange reagent in PBS for a 5 min period of irradiation with UV-B (312 nm, 5 mJ/cm^2^; TF-20M; Vilber Lourmat, Marne la Vallée, France) followed by incubation at 37°C for 30 min. The cells were observed using a Leica AF 6000 LX fluorescence microscope system (Leica Microsystems, Leica, Wetzlar, Germany). Fluorescence signal intensity was calculated by ImageJ software (Wayne Rasband, NIH) as previously described.^(11,12)^ Cells were also harvested by trypsin treatment following washing with PBS (two times) for cell cytometry analysis; harvested cells were resuspended in DMEM. The cell samples (25 µl) were loaded into the half moon-shaped sample loading areas of Tali Cellular Analysis Slide (Thermo Fisher Scientific). They were examined by a Tali image-based cytometer (Life Technologies), which is a 3-channel (bright field, green fluorescence, and red fluorescence) benchtop cytometer. CellROX^+^ ratios in EGFP^+^ cells were calculated as oxidative damaged cell ratios.

### Statistical analysis

Statistical differences were determined by the two-sided Mann-Whitney’s *U* test. Differences with *p*<0.05 were considered significant. All data in graphs are presented as means ± SEM.

## Results

### Expression and localization of PRDX4t-EGFP in HEK293T cells

**Fig. 1:**
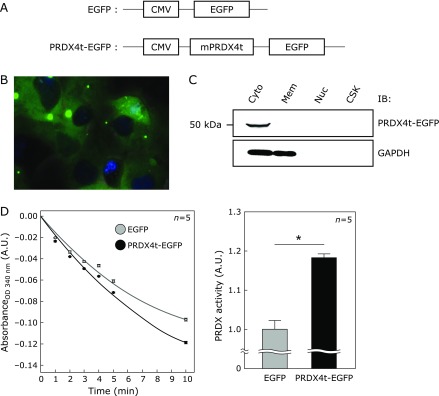
PRDX4t-EGFP expression and localization in HEK293T cells. (A) PRDX4t-EGFP and EGFP constructs. (B) A representative image of HEK293T cells ectopically expressing PRDX4t-EGFP is shown. The representative fluorescence image was taken using a fluorescence microscope system (Leica). PRDX4t-EGFP, green. (C) Cytosol (Cyto), membrane and membranous organelles (Mem), nucleus (Nuc), and cytoskeleton (CSK) extracts were prepared from PRDX4t-EGFP-transfected HEK293T cells separated by SDS-PAGE. PRDX4t-EGFP protein was monitored by immunoblot. (D) Time course for degradation of absorbance of NADPH (left) and the mean of quantitative PRDX activity from the slope (right) in EGFP or PRDX-EGFP expressed cells is shown. *P* values were derived from two-sided Mann-Whitney’s *U* test (*p<0.01). A.U., arbitrary unit. Data are means ± SEM; *n* = 5.

To determine the localization of PRDX4t in mammalian cells, we first tried to express PRDX4t in the mammalian cell line HEK293T. We prepared a PRDX4t-EGFP fusion construct (pEGFP-N1-PRDX4t) and an EGFP control plasmid (pEGFP-N1) for transfection assays (Fig. 1A). Typically, endogenous mouse PRDX4t has been shown to localize only to the cytosol of testicular cells.^(13)^ Consistent with this previous report, we observed the expression of PRDX4t-EGFP in the cytosol of HEK293T cells using a fluorescence microscope system (Fig. 1B). PRDX4t-EGFP protein localization was also determined by immunoblot after cell fractionation and PRDX4t-EGFP was present only in the cytosolic fraction (Fig. 1C). In addition, we found that the PRDX activity of PRDX4t-EGFP expressed cells was significantly high compared with that of the control cells (Fig. 1D). These results indicate that the PRDX4t-EGFP expressed cells may serve as a model for facilitating the understanding of the antioxidant function of PRDX4t in mammalian cells.

### PRDX4t-expressing cells showed lower ROS levels compared to control cells

**Fig. 2:**
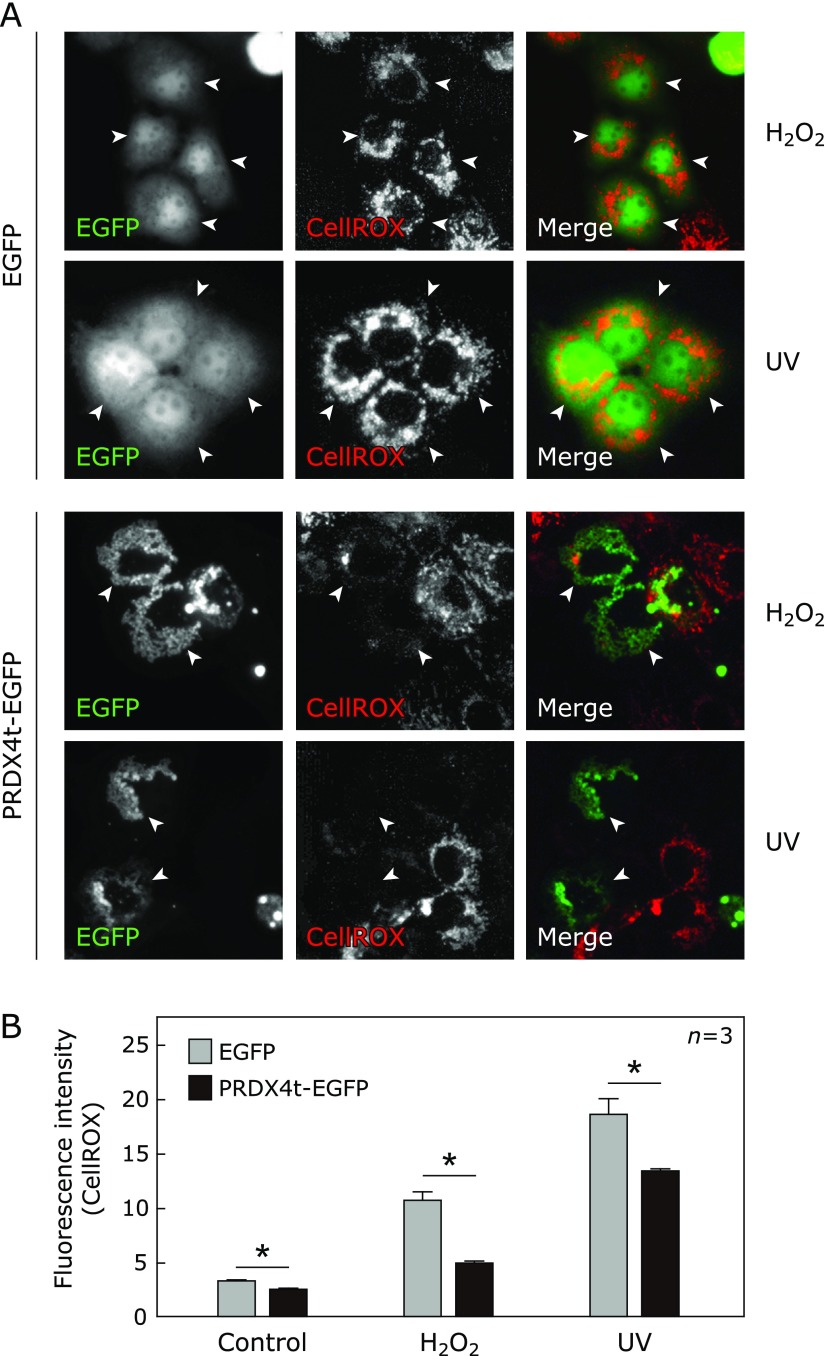
Intracellular ROS detection in HEK293T cells expressing PRDX4t-EGFP. (A) HEK293T cells expressing PRDX4t-EGFP or EGFP were stimulated with 250 µM H_2_O_2_ treatment or 5 min UV-irradiation. These fluorescence images were taken using a fluorescence microscope system (Leica). PRDX-EGFP or EGFP normally expressing cells were pointed by white arrow. (B) Quantitative analyses of fluorescence intensity are shown. The CellROX fluorescence intensities were measured as ROS levels in the cells expressing PRDX4t-EGFP or EGFP. Gray and black bars indicate the cells expressing EGFP and PRDX4t-EGFP, respectively. *P* values were derived from two-sided Mann-Whitney’s *U* test (*p<0.05). Data are means ± SEM; *n* = 3.

Since it has been reported that the overexpression of antioxidant enzymes protects cells against oxidative stress,^(14–16)^ we examined whether PRDX4t-transfection increases the antioxidant ability of the cells. In this investigation, we performed fluorescence microscopy after the PRDX4t-EGFP-CellROX combination method. As a result, we observed that cells expressing PRDX4t-EGFP demonstrated decreased CellROX fluorescence compared to cells just expressing EGFP (Fig. 2A). Quantification of CellROX fluorescence revealed that PRDX4t expression contributes to the cellular antioxidant ability after oxidative stress, even in untreated control cells (Fig. 2B). Therefore, we were able to demonstrate that PRDX4t can actually play a protective role against oxidative stress in mammalian cells.

### Cytometrical analysis indicated that PRDX4t plays a protective role against oxidative stress caused by H_2_O_2_ treatment or UV-irradiation

**Fig. 3:**
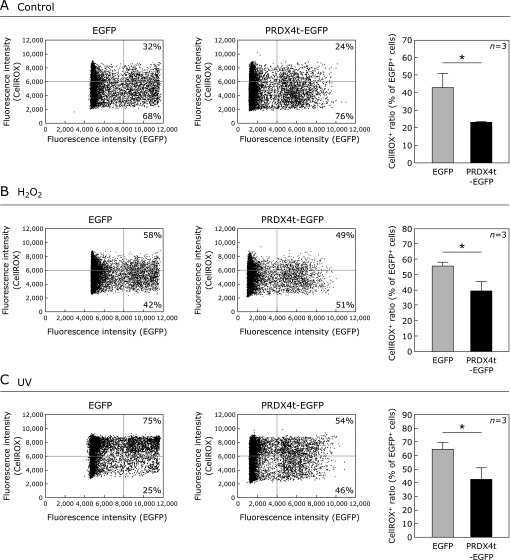
Image-based cytometry analysis of HEK293T cells transfected with the PRDX4t-EGFP. (A–C) The representative plots of the analyzed data in HEK293T cells transfected with EGFP plasmid (left panel) or PRDX4t-EGFP plasmid (right panel). The EGFP^+^ and CellROX^+^ areas were gated by the dotted line at appropriate fluorescence densities. Histograms show the quantification of CellROX^+^ ratios (% of EGFP^+^ cells). Cells were untreated controls (A) or treated with H_2_O_2_ (B) or UV (C) before analysis. Gray and black bars indicate the cells expressing EGFP and PRDX4t-EGFP, respectively. *P* values were derived from two-sided Mann-Whitney’s *U* test (*p<0.05). Data are means ± SEM; *n* = 3.

We quantified the antioxidant effect of PRDX4t in HEK293T cells using an image-based cytometer after treatment with 250 µM H_2_O_2_ or UV (312 nm)-irradiation for 5 min. Cells, which were mainly transfected with PRDX4t-EGFP or the EGFP control plasmid, were located in the right field on the panels (EGFP^+^ cells; Fig. 3). The histograms indicate oxidatively damaged cell ratios, calculated as average CellROX^+^ ratios in the EGFP^+^ cells. Interestingly, the percentage of oxidatively damaged fractions of PRDX4t-EGFP-expressing cells was lower than the percentage of EGFP-expressing cells, even in the untreated condition (Fig. 3A). Consistent with microscopic observations, PRDX4t-EGFP-expressing cells showed high resistance against oxidative stress after H_2_O_2_ treatment or UV-irradiation compared to EGFP-expressing cells (Fig. 3B and C).

## Discussion

PRDX4t, a newly described member of the PRDX family, is specifically expressed in testicular cells.^(13)^ PRDX4 gene knockout mice, in which PRDX4t expression is decreased compared to wild type mice, show increased spermatogenic cell death due to oxidative stress.^(7,10)^ However, the antioxidant function of PRDX4t against oxidative stress has not yet been evaluated. In the present study, the overexpression of PRDX4t protected HEK293T cells from H_2_O_2_- or UV-induced oxidative stress as determined by image-based cytometer analysis (Fig. 3). These results are compatible with the typical enzymatic function of PRDX in antioxidant defense. This study further evaluated the antioxidant function of PRDX4t by fluorescence microscopy. Consistent with image-based cytometer results, we determined that PRDX4t-expressing cells achieve a higher resistance against oxidative stress than control cells (Fig. 2). In the two image-based analysis, especially in Fig. 3A, we detected that overexpression of PRDX4t suppresses oxidative stress in cultured cells even in control condition. We considered that this was caused by stress accumulation during experiment operation and *in vitro* culture stress because of ambient 21% oxygen. Moreover, we observed higher PRDX activity in PRDX4t overexpressed cells than in control cells (Fig. 1D). These results indicate that PRDX4t plays a protective role against oxidative stress in mammalian cells.

Generally, mammalian PRDXs are classified as six isoforms.^(3)^ These PRDXs are thought to have Trx-dependent peroxidase activity, in which H_2_O_2_, as well as a wide range of organic hydroperoxides (ROOH), are reduced and detoxified.^(2)^ Interestingly, previous studies have demonstrated that typical 2-Cys PRDXs, which include mammal PRDX1–4, play as regulators of H_2_O_2_-sensing cellular signaling.^(5,6,17,18)^ PRDX4t may play another role, such as in the signal regulation of mammalian cells; therefore, further studies are needed to evaluate the function of PRDX4t, which is distinct from the antioxidant behavior in mammalian cells.

Intrinsically, PRDX4t is specifically expressed in sexually matured testes. In the testes, spermatogenic cells experience dramatic changes in the gene expression, morphogenesis, and redox environment during spermatogenesis. Although the chromatin of somatic cells is constituted by histones in ordinary somatic cells, sperm nuclear histones are replaced by protamines during the spermatogenic process.^(19)^ The protamines of primates and rodents contain multiple cysteine residues that are oxidized to form disulfide bridges (sulfoxidation) that contribute to resistance against oxidative stress in the chromatin of spermatogenic cells; PRDX4t is thought to play an important function as a sulfoxidase during spermatogenesis.^(7,20)^ This sulfoxidase function of PRDX4t is similar to the protein folding function of PRDX4 localized to the ER/Golgi apparatus.^(21)^ In summary, we showed possibility that PRDX4t may not only function as a sulfoxidase but also may have an antioxidant function in spermatogenic cells during spermiogenesis.

## Abbreviations

CO_2_: carbon dioxide
DMEM: Dulbecco’s modified Eagle medium
ER: endoplasmic reticulum
FBS: fetal bovine serum
H_2_O_2_: hydrogen peroxide
PRDX: peroxiredoxin
PVDF: polyvinylidene fluoride
ROS: reactive oxygen species
Trx: thioredoxin
